# Mr. Buckston Browne's Views

**Published:** 1902-02-22

**Authors:** 


### MR. BUCKSTON BROWNE'S VIEWS.

We have just received a reprint of the " Harveian Lectures on Twenty-five Years' Experience of Urinary Surgery in England," by Mr. Buckston Browne, in which this subject is dealt with, and while we are on the topic we may as well quote what he says about it. He says that large masses of the prostate have been successfully removed ; " some surgeons have, indeed, successfully scooped out such very large masses entire that they have thought that the whole prostate has been removed." But surely, he adds, such a phrase as " total extirpation of the prostate " is misleading. " The prostate can no more be extirpated without the use of the knife than can a piece of intestine." " It is quite clear, I think, that the phrase 'total extirpation of the prostate' has been used in error and by surgeons who have been fortunate in having only met with the simpler or adenomatous form of prostatic enlargement, where large masses are easily shelled out." Which is all very well, but what is the " capsule " which, as Dr. Freyer shows, encloses the masses which he removes ? We do not think it is altogether creditable to British surgery that a dispute of this kind should go on. If it is a mere question of anatomy, that is of fact, which is capable of being solved at once and authoritatively by a couple of mornings' work in the post-mortem room, it is undoubtedly wrong that the wrangle should continue.

